# The effects of acute exercise on long-term episodic memory: a systematic review and meta-analysis

**DOI:** 10.3389/fcogn.2024.1367569

**Published:** 2024-04-04

**Authors:** Ahmed S. Qazi, Daphne Schmid, Nicole Gridley, Kate Lambourne, Andrew J. Daly-Smith, Phillip D. Tomporowski

**Affiliations:** ^1^Department of Kinesiology, University of Georgia, Athens, GA, United States; ^2^Carnegie School of Education, Leeds Beckett University, Leeds, United Kingdom; ^3^ICF Inc., Fairfax, VA, United States; ^4^Faculty of Life Sciences and Health Studies, University of Bradford, Bradford, United Kingdom; ^5^Centre for Applied Education Research, Wolfson Centre for Applied Health Research, Bradford Royal Infirmary, Bradford, West Yorkshire, United Kingdom

**Keywords:** episodic memory, memory encoding, long-term memory, acute exercise, free-recall, recognition, cued-recall

## Abstract

Research findings reveal a relationship between acute bouts of exercise and procedural/declarative memory. Prior systematic reviews report small/moderate effects of acute exercise on episodic long-term declarative memory. A somewhat overlooked issue is the influence of exercise on specific types of episodic memory processing. The primary focus of this systematic review and meta-analysis was to evaluate the effects of acute bouts of exercise prior to, during, and following encoding on free-, cued-recall, and recognition episodic memory. PubMed, Scopus, and EBSCO databases were entered, and 42 experiments were subject to meta-analysis. Exercise prior to encoding improved memory (d = 0.23) and affected free-recall (d = 0.40) tests of memory more than cued-recall (d = 0.08) or recognition (d = −0.06) memory. Exercise following encoding improved memory (d = 0.33) and affected recognition (d = 0.62) memory more than free- (d = 0.19) or cued-recall (d = 0.14) memory. Exercise during encoding did not influence memory (d = −0.04). Moderator analyses revealed that exercise before encoding impacted memory differentially on the basis of age, exercise type, and test-timing. When exercise occurred after encoding, age and exercise type, but not timing of the test influenced memory performance. Exercise before and after encoding has selective effects on episodic memory. Additional experiments that evaluate how bouts of exercise influence memory encoding are warranted.

**Systematic review registration:**

PROSPERO, identifier CRD42020202784.

## 1 Introduction

There has been a marked increase in recent years in studies of the effects of individual bouts of exercise on long-term memory. Much of this research activity was spurred by several literature reviews that provide clear support for the view that acute exercise enhances memory (Lambourne and Tomporowski, 2010; Roig et al., 2013). Reviewers conclude that the strength of the acute exercise-memory relation is moderated by such characteristics as the type of exercise performed and its intensity and duration, the temporal relation between exercise and encoding, and the delay between encoding and testing. Questions remain, however, concerning the degree to which the impact of exercise depends on the manner in which memories are processed, stored, and retrieved. Answers to these questions have implications for both researchers and practitioners who are interested in designing exercise or broader physical activity interventions that can maximally affect memory and learning.

A meta-analytic review of the exercise-memory literature conducted by Roig et al. (2013) categorized memory tasks in terms of short-term and long-term memory tests. Studies were classified on the basis of the time between encoding and testing, with delays longer than 2 min categorized as long-term memory. Further, long-term memory tests were divided into declarative (episodic or semantic) and non-declarative (procedural) memory tests. The results of seven experiments indicated that acute bouts of exercise enhanced performance on long-term procedural and declarative memory tests, with moderate to large effect sizes. More recently, Loprinzi et al. (2019a) focused specifically on the effects of acute bouts of exercise on short-term and long-term episodic memory. The authors' review of 25 experiments provided information concerning the temporal relation between exercise bouts and encoding (prior, during, and following encoding) and such study characteristics as exercise type, intensity, and duration. Exercise performed prior to and following encoding enhanced performance, with larger effect sizes when exercise was performed following encoding than prior to encoding. Exercise performed during encoding had a negative effect on episodic memory.

The meta-analytic reviews by Roig et al. (2013) and Loprinzi et al. (2019a) and others (Lambourne and Tomporowski, 2010) support a causal relation between acute exercise and memory storage. These reviews also clarify exercise parameters and individual difference factors that influence the strength of the relation. Yet to be addressed, however, are questions concerning the uniformity of acute exercise effects across different forms of memory. The memory tests employed in experiments evaluated in previous reviews vary widely and the cognitive processes involved in performing individual tests cannot be clearly delineated. Roig et al. (2013), for example, included tests of verbal fluency, associative memory, paragraph reading, code-substitution, object recognition tasks, and an assortment of word recall tests. Similarly, Loprinzi et al. (2019a) included studies that employed the California Verbal Learning Test, Rey Auditory Verbal Learning Test (RAVLT), paragraph recall, image recognition, recall of filmed scenarios, and laboratory constructed tests. The consensus among re-searchers is that humans possess several different types of memory, all of which are maintained via underlying brain networks (Schacter et al., 1999; Squire and Wixted, 2011; Eichenbaum, 2017; Loprinzi et al., 2017) and are controlled by cognitive processes that are involved in encoding and retrieval (Wilson and Criss, 2017).

The present systematic review was designed to provide clarity concerning the effects of exercise on long-term memory by focusing on episodic memory, a type of declarative memory that is involved in storing information about temporally arranged or dated events. Acute exercise experiments typically involve asking participants to study material (e.g., words) in conjunction with a bout of exercise. Episodic memory is typically measured via tests of recognition, cued-recall, or free-recall. Recognition tests are characterized by methods in which a participant is asked to identify whether or not a specific item (e.g., word) was previously presented. Cued-recall tests often involve the presentation of pairs of items (e.g., word pairs), one of which is to be remembered while the other serves as a priming stimulus that assists in the reintegration of the targeted item. Free-recall tests require an individual to remember target items without the aid of cues. Recognition memory test performance is typically considered easier than cued- and free-recall test performance because the participant is not required to generate a memory of the item and then determine if it was previously seen (Malmberg et al., 2019).

**Figure 1:**
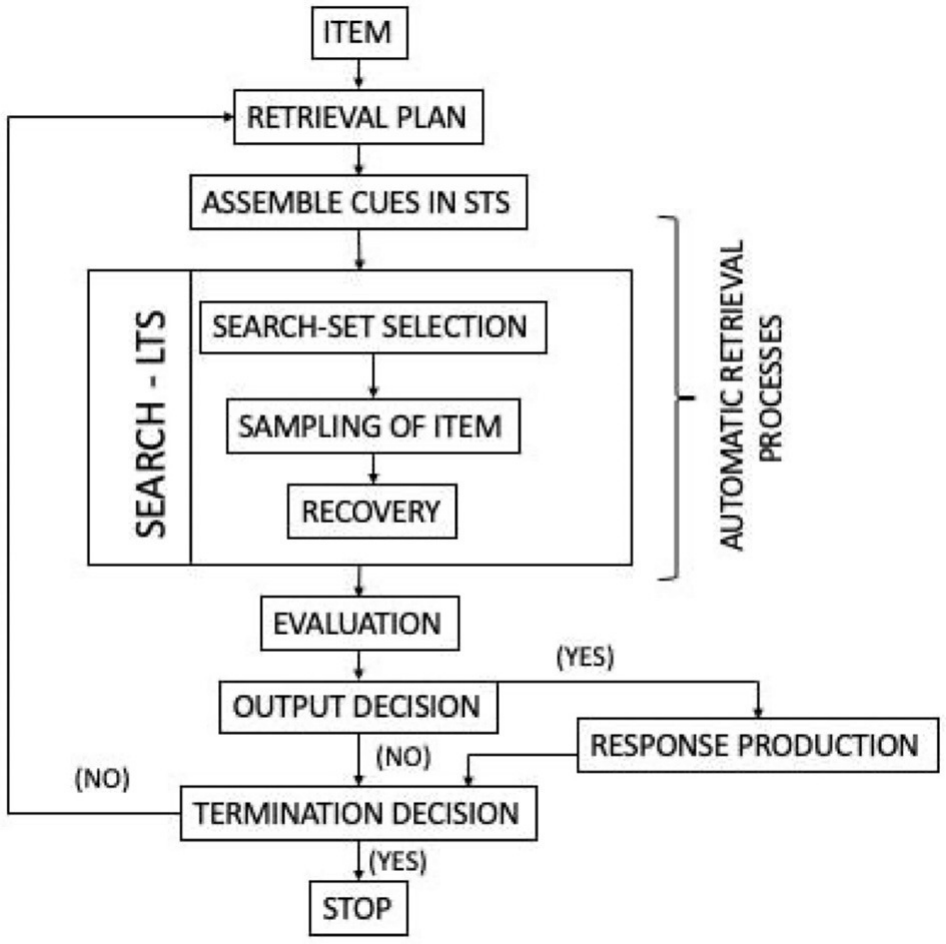
The Search of Associative Memory (SAM) model. A generalized depiction of the various phase of retrieval (STS, short-term search; LTS, long-term search). Modified from Raaijmakers and Shiffrin (1981).

**Figure 2:**
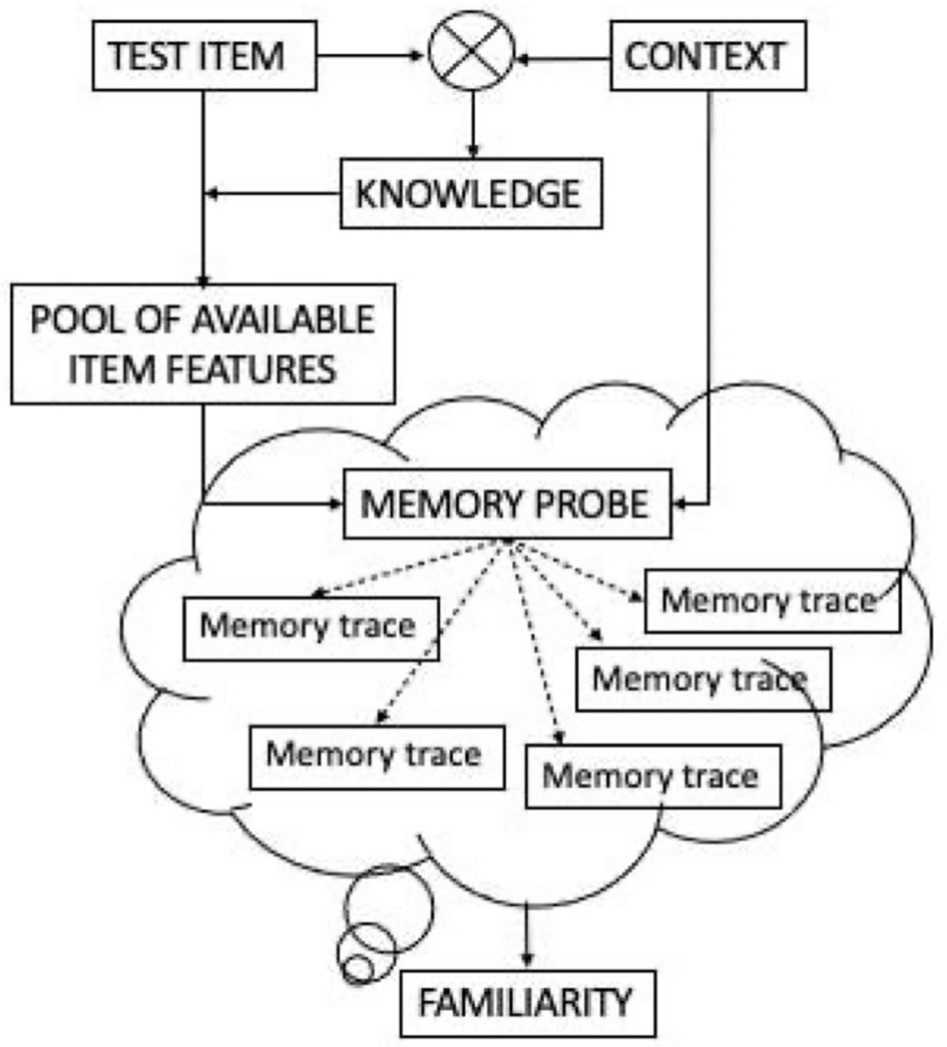
The Retrieving Effectively from Memory (REM) model. A schematic depiction of the recognition process. A memory probe is formed from a combination of the current context and both physical and semantic features of the test item. Item features are gradually sampled into the probe which is continually compared in parallel to all the traces in memory, each of which is activated to a degree depending on the similarity between features in the probe and those in the trace. To make a recognition decision, a participant tracks changes in the overall level of activation of these traces – termed “familiarity – from an initial level determined by context alone.” Modified from Cox and Shiffrin (2017).

Numerous psychological theories of memory have been proposed to explain how sensory experiences are manipulated and stored in memory and how stored memories are retrieved (Malmberg et al., 2019). An important contribution to memory research was a model of memory processing introduced by Atkinson and Shiffrin (1968, 1971). Central to their theory was conceptualization of the processes of a short-term memory buffer and its relation to long-term storage and retrieval. Theory-based experimentation over the course of five decades led to several modifications and an eventual reconceptualization of the processes involved in recall and recognition memory. The Search of Associative Memory (SAM) (Figure 1) (Raaijmakers and Shiffrin, 1981; Gillund and Shiffrin, 1984) and Retrieving Effectively from Memory (REM) (Figure 2) (Cox and Shiffrin, 2017) models emerged; they continue to guide contemporary memory research (Malmberg et al., 2019). As shown in Figure 1, the SAM model of episodic memory is based on the assumption that experienced events are held in short-term memory where rehearsal processes create separate memory traces that strengthen as new information is added. Retrieval consists of a search process in which multiple traces are sampled; selection is based on trace strength. Long-term forgetting is thought to be due to interference and search termination rather than a function of a decay in trace strength. Central to the SAM model is the prediction that memory retrieval reflects a dual-coding process that includes memories of the items to be re-membered and memories of the environmental context present when items are studied. During encoding, the environmental setting provides “background” information (e.g., location, mood, temperature, arousal) that is not experimentally varied and is not the focus of the experiment. Recall and recognition memory performance reflects the combination of the strength of individual memory traces for items and for context. As shown in Figure 2, the SAM-REM model makes explicit assumptions concerning strengths of item and context memory and how trial-to-trial variations (e.g., list length, item similarity) create “noise” that affects memory retrieval (Criss and Shiffrin, 2004). The environmental context in which items are encoded contribute to how well memory search leads to an appropriate match. Retrieval from long-term memory involves sampling of memory traces and a reconstructive process that involves probabilistic judgements based on the relevance of sample traces (Criss and Shiffrin, 2004).

The SAM-REM models describe different processes for free-recall and recognition. For free recall, there is a competition among memory traces; selection is based on how well an internally generated context cue matches the context stored in a trace. Episodic recognition memory does not require the generation of past episodic details from memory. All that is required is to distinguish an item that is presented during the context of encoding from foils that were not presented. Free recall is often described synonymously as remembrance, whereas recognition reflects familiarity. Confusion during recognition memory is created by noise associated with items experienced in different environmental contexts.

Acute bouts of exercise are hypothesized to produce systemic changes in physiological arousal and alterations in neural noise that affect neural networks involved in executive processing and mental performance (McMorris, 2016a) and memory storage (McGaugh, 2015; Meeusen et al., 2017). Several narrative and systematic reviews conclude that acute bouts of exercise affect working-term memory processes and decision-making speed (Tomporowski, 2003; Lambourne and Tomporowski, 2010; Chang et al., 2012; McMorris and Hale, 2015). The physiological changes that occur during a bout of exercise may also influence the strength of item and context memory traces stored in memory, which would facilitate memory retrieval. Several neurophysiological mechanisms have been identified that may explain how acute exercise improves memory processes. Functional changes in neurotransmission, neurotrophic factors (e.g., BDNF and vascular endothelial growth factor), increased blood flow, receptor activity, and glucose/oxygen metabolism may ensue as a result of acute bouts of exercise (Loprinzi et al., 2017; El-Sayes et al., 2019; Loprinzi, 2019). Increases in neurotrophic and growth factors have been shown to improve cellular processes that lead to structural changes that are correlated with behavioral change. Neurophysiological adaptations may improve the encoding, storage, and retrieval of memory items. Given the evidence for the existence of different types of memory stores, which differ in their underlying mechanisms, it is of theoretical and practical interest to determine whether acute exercise has a global effect on all types of memory processing, or whether the effects are selective.

The standard view of salutary interventions, such as physical activity and exercise, is that they promote global brain health, which leads to gains in cognition (Hillman et al., 2008). Theory-based research conducted over the past decade on the neurobiology of memory provides evidence to suggest that environmental and contextual factors exert selective effects on episodic memory. Reviews of experiments that examine both behavioral and neurophysiological outcomes reveal that contextual manipulations differentially influenced free-recall and recognition memory (Eichenbaum et al., 2007; Yonelinas et al., 2019). The pattern of results obtained under contextual conditions present during encoding (e.g., study time, elaboration, attention, and rote repetition) and retrieval (e.g., attention and test delay), are explained in terms of specific neurological structures and networks. Tests that involve the recollection of items are dependent on hippocampal structures while tests of familiarity and recognition depend on parahippocampal structures and the entorhinal cortex (Brickman et al., 2014). The double anatomical dissociation observed in clinical studies conducted with individuals with amnesia and laboratory experiments conducted with humans, monkey, and rats indicate that contextual factors may differentially impact underlying neural networks that are involved in episodic memory (Yonelinas et al., 2019). Such studies provide clear evidence that there exists a distinct double dissociation among several brain structures responsible for the consolidation of long-term memory. This double dissociation between the perirhinal cortex and hippocampus has been implicated among animal models to produce differential outcomes with respect to recognition, spatial, and recall memory (Winters et al., 2004). Given that acute bouts of physical activity impact episodic memory similarly to other contextual contexts, it will shed light on the neuroanatomical pathways that impact memory storage and retrieval as well as help predict the effectiveness of specific educational methods designed to benefit long-term memory.

The present review extends previous literature analyses (Roig et al., 2013; Loprinzi et al., 2019a) by focusing on the effects of acute bouts of exercise performed before, during, and after encoding on specific types of episodic memory processes described in contemporary psychological theory and neurobehavioral hypotheses.

## 2 Materials and methods

The primary aim of the present paper was to conduct a systematic review and meta-analysis to assess the impact of acute bouts of exercise on episodic memory. The systematic review protocol was registered with International Prospective Register of Systematic Reviews on 27th August 2020 (CRD42020202784) and adheres to the Preferred Reporting Items for Systematic Review and Meta-Analysis Protocols (PRISMA-P). We undertook a two-stage search process to identify relevant articles for the meta-analysis; first, a search of scientific databases, and then a manual search of the reference lists of included studies for additional papers not previously identified. We also conducted a follow-up search procedure in February 2023 to identify any additional references that were subject to our systematic review. The secondary search methods were identical to the primary search. These methods for systematic review and meta-analysis were in line with those used previously (Siddaway et al., 2019).

**Figure 3:**
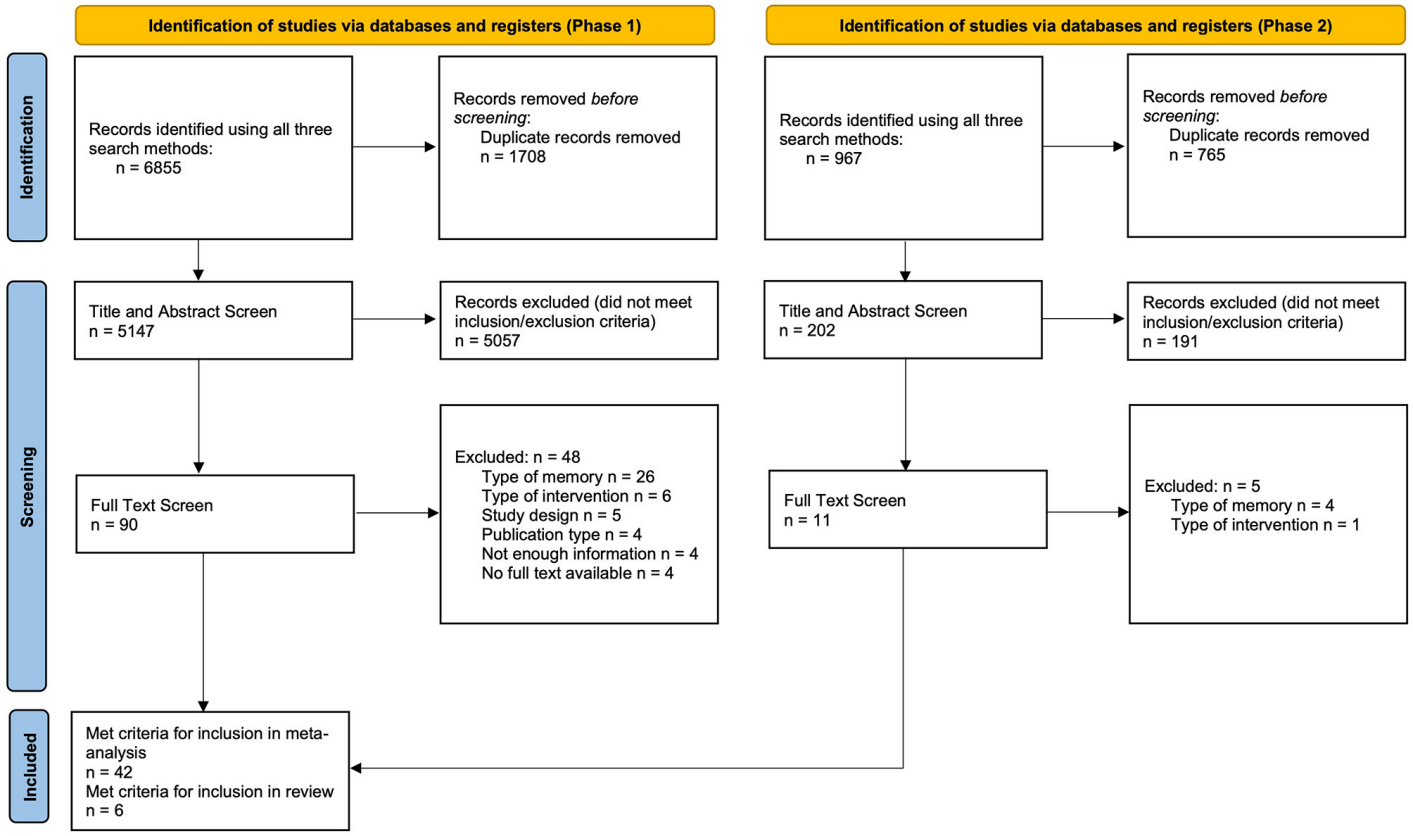
PRISMA flow diagram illustrating retrieval and inclusion in meta-analysis.

Three commercial platforms (Pubmed, Scopus and EBSCO) hosting six scientific databases (CINAHL Complete, ERIC, MEDLINE, APA PsycArticles, APA PsycInfo, SPORTDiscus with Full Text) were searched in March 2020 and then once again in November 2020. Databases were searched in English. The PRISMA flow diagram illustrating retrieval is presented in Figure 3. A detailed flow diagram is presented in the Supplementary material. Following searches, reference lists of identified articles and previous systematic reviews were reviewed to identify further relevant studies. As shown in Figure 3, the database search identified 6,855 references. In 1,708 duplicates were identified and removed, leaving 5,147 references for screening. The first screening phase (titles and abstracts) yielded 90 references for full text screening after 5,057 references were excluded. Of these, 48 were excluded, leaving 42 to meet our inclusion criteria. The review considered studies published in English; no date limits were set. Both control trials and cross-over trials were included. Studies were excluded when they did not meet the key inclusion criteria described below. Gray literature was not consulted, on the basis that most rigorous studies will include peer-reviewed consideration. The secondary search resulted in 967 references. Seven hundred and sixty five duplicates were identified and removed, leaving 202 for full text screening. The initial screening phase (title and abstract) yielded 11 references for full text review. Following the full text review, 6 references met our inclusion criteria and were included in the secondary analysis.

### 2.1 Inclusion/exclusion criteria

The keyword search strategy was iteratively developed via the author team. Key search terms and their derivatives were pooled in two key themes related to memory and the mode of exercise prior to being combined for the final searches. The population specified human participants aged 5 years or older. The environment was not specified to enable a broad array of studies in both lab and field-based settings. The intervention type was an acute bout of exercise before, during, or after an encoding task, that may be a repeated design within a day or in a cross-over condition. The exercise modes were developed through an initial review of the literature to identify all of the acute exercise modes. The outcome measures were aligned to memory types (long-term declarative memory, semantic memory, episodic memory, and spatial memory) and memory assessment (free-recall, cued-recall, recognition, paired-associate, object location, sentence recall, and word fluency). Exclusion criteria was opposite to the inclusion criteria, in addition to the studies included; participants who reported suffering from a concussion or similar head trauma within the past 6 months, studies on participants prescribed psychotropic medications, a condition that influences memory (e.g., learning disorders, developmental disorders, specific clinical diagnosis based on memory disorders), studies focused on specific health conditions (e.g. cancer, diabetes), no assessment of memory retention over time, and studies that used the following designs: cross-sectional studies, longitudinal studies, or chronic studies (more than one bout of exercise over an extended period of time or extending two or more days).

### 2.2 Article screening and data extraction

All articles retrieved as part of the scientific platform search were downloaded into an Endnote database and duplicates were removed. One author (NG) performed a title and abstract screen while a second author (AD-S) performed a random check of 20% of the articles. Before performing a full-text screen, the inclusion/exclusion criteria outlined above was applied. All authors performed full text screening of articles that met the initial criteria. Once the final eligible studies were identified, a second search of the literature was conducted by assessing the accompanying reference sections. The reference sections were manually searched, cited articles were extracted by hand, and the article name, authors and date of publication were inserted into an Excel spreadsheet. These extractions were cross checked against the original Endnote database, duplicates were identified and removed, and all titles and abstracts were assessed prior to proceeding to a full-text screen by all authors using Google Scholar. Several discrepancies in the application of selection criteria were identified within the selection process but were resolved through consensus with all co-authors. Study characteristics, effect size data, and data for moderator variable coding were extracted from all eligible articles. At the onset of coding, approximately 10% of articles were double–coded and any inconsistencies were discussed. Upon consensus between all authors, the remaining articles were single-coded by KL and cross-checked for accuracy (PT and AQ). Data extracted to an Excel spreadsheet included author names, publication year, study design, sample type and characteristics (gender, age, sample size), memory test characteristics (timing, type), exercise protocol (type, mode, intensity, length), and results.

The screening and data extraction process for the secondary search was identical to the primary search. Two authors (AQ and DS) performed a title and abstract screening. Similar to the primary search, study characteristics and effect size data were extracted from all articles. All data was cross checked for accuracy (AQ and DS) and any discrepancies were resolved through consensus.

### 2.3 Assessment of study quality

Consistent with PRISMA-P guidelines, two independent reviewers (NG and AD-S) assessed overall and subdomain risk of bias using a modified version of the Downs and Black (Downs and Black, 1998) checklist for methodological quality for both randomized and non-randomized studies of health care interventions (Daly-Smith et al., 2018). The use of the modified criteria reflected the need for a risk-of-bias tool that was specific to acute experimental studies. Study authors further developed the criteria to address specific issues discussed in previous reviews (online Supplementary file 2). Standardization was used on three papers. The remaining papers were divided equally between the two authors for assessment. A 20% sample of both authors' assessment was moderated by the other reviewer. Disagreements between re-view authors over the risk of bias were resolved by discussion, with a review of the specific criteria being performed on all non-moderated papers.

Assessment of study quality for the secondary search was identical to the primary search and was conducted independently by two authors (AQ and DS). Any disagreements between the authors were resolved via discussion.

### 2.4 Meta analytic methods

To assess the effect of acute exercise on different encoding paradigms (encoding before exercise, during exercise, or following exercise), three separate meta-analyses were conducted. Meta-analyses were conducted using Comprehensive Meta-Analysis CMA software (Version 3 for Windows, Biostat company, Englewood NJ, USA). CMA enables the harmonization of data presented in different formats and from different study designs (i.e., between-subjects, within-subjects). Cohen's d was calculated when means, standard deviations, and sample sizes were available. When these data were not available, Cohen's d was estimated using *t* or *p* values and sample size information. Cohen's d was used to control for Type I error rates (Huedo-Medina et al., 2006). Effect sizes were calculated such that a beneficial effect of acute exercise on long-term memory was indicated by a positive effect size. The degree of heterogeneity of the effect sizes was evaluated with the Cochran's Q-statistic. Egger's regression test was used to evaluate the potential publication bias.

Categorical moderators were determined a priori and were chosen based on logical, theoretical, and previous empirical relations between acute exercise and episodic memory. Similar to the previous meta-analysis by Loprinzi et al. (2019a), cardiorespiratory fitness was not examined because few studies reported the measure. The acute exercise paradigm was coded as exercise prior to encoding, exercise during encoding, and exercise following encoding. Episodic memory type was coded as free-recall, cued-recall, and recognition. The timing of the memory test was coded as same day, 1 day later, 2 days later, 1 week later, and 2 weeks later. Age was categorized as elementary (6–13 years), high school (14–17 years), young-adult (18–24 years), adult (25–44 years), middle-age (45–60 years), and older adults (>60 years) (Roig et al., 2013; Loprinzi et al., 2019a). Sex was coded as males, females, mixed, predominately male (>70% of sample), or predominantly female (>70% of sample). For the exercise protocol, exercise was coded by type (aerobic, anaerobic, or muscular resistance), mode [running/walking, cycling, other (e.g., circuit training, resistance training, stepping)], duration (very short = 10 min or less, short = 11–20 min, medium = 21–39 min, long ≥ 40 min), and intensity (low, low to moderate, moderate, high) (Garber et al., 2011; Roig et al., 2013). Note that many different metrics for exercise intensity were used (RPE, HRR, max HR, etc.) which made it difficult to apply standard thresholds such as those suggested by the American College of Sports Medicine. The author's descriptor of the intensity level was used when provided; when it was not stated we used our expert judgement. Moderators also included study quality (excellent, good, fair, or poor) (O'Connor et al., 2015) and study design (between-subjects, within-subjects).

Moderators were tested using Q with a mixed effects analysis. Heterogeneity was determined by Cochrane's Q statistic and I^2^ values, whereby values of < 25, 50, and 75 were considered to indicate low, moderate, and high levels of heterogeneity, respectively (Higgins et al., 2003). Subgroup moderator analyses were conducted for the encoding paradigms if I^2^ values demonstrated at least moderate heterogeneity.

## 3 Results

### 3.1 Articles included in analyses

Using the predefined search strategies, three stages of article searches were conducted. At the conclusion of the first stage, 23 articles were considered eligible for inclusion in the final meta-analysis. At the conclusion of the second stage, 12 articles were deemed eligible for inclusion in the final meta-analysis. At the conclusion of the third stage, 7 articles were considered eligible for inclusion in the final meta-analysis.

In total, 42 articles met the inclusionary criteria and were used for quantitative analyses in the present meta-analytic review. A detailed visual representation of the retrievals at each stage of the review is summarized in the flow diagram in Appendix A. Table 1 provides a summary of the included experiments.

**Table 1:** Summary characteristics of experiments included in meta-analysis.

**References and Country**	**Participant characteristics**			**Exercise intervention**				**Study design**		**Type of memory assessed**	**Memory assessment**	**Time between encoding and recall/ recognition**
	**N**	**Age**	**Sex**	**Duration**	**Intensity**	**Mode**	**Control**	**Temporal period**				
Amico and Schaefer (2020) (US)	78	31.03 (3.6)	39 F 39 M	-	Mod	Running, Dribbling	Standing	During	Between	Cued recall (words)	Vocabulary learning task	Immediate 24 h
Austin and Loprinzi (2019) (US)	20	21.6 (0.7)	50% F 50% M	10 mins	-	Treadmill	Sudoku	Before	3-arm Within subjects	Free recall (words)	RAVLT	10 mins
Coles and Tomporowski (2008) (US)	18	22.2 (1.6)	NK	40 mins	Mod	Cycle ergometer	Rest watching documentary	Before	Within subjects	Free recall (words)	Visual free recall test	100 seconds 12 mins
Frith et al. (2017) (US)	88	21.9 (2.4)	45.5% M 54.5% F	15 mins	High	Treadmill	Sitting	Before During After	Between subjects	Free recall (words)	RAVLT	20 mins 24 h
Haynes and Loprinzi (2019) (US)	24	20.9 (1.9)	66.7 F	15 mins	Mod	Treadmill	Rest for 5 mins	Before During After	Within subjects	Free recall and recognition (words)	RAVLT	20 mins 24 h
Delancey et al. (2019) (US)	40	Exp – 21.9 (0.6) Cont – 20.8 (0.3)	~50% F ~50% M	15 mins	Low Mod High	Treadmill	No exercise	After	Between subjects	Free recall (words)	RAVLT	24 h
Dilley et al. (2019) (US)	60	20.8	Cont – 80% F Mod – 95% F High – 95% F	15 mins	Mod High	Treadmill	Sudoku	Before	Between subjects	Cued-recall and Recognition (words)	DRM Paradigm	Immediate 10 mins
Etnier et al. (2014) (US)	43	11-12	28 F 15 M	To exhaustio n	High	PACER Test	No treatment control	Before	Between subjects	Free recall and recognition (words)	RAVLT	2 mins 24 h
Hötting et al. (2016) (Germany)	81	22 (2.36)	41 M 40 F	To exhaustion	Low High	Cycle ergometer	Relaxing	After	Between subjects	Cued recall (words)	Polish-German Vocab test	20 mins 24 h
Johnson et al. (2019) (US)	24	21.5 (1.2)	54.2% F 45.8% M	10 mins	Mod	Sprints on indoor court	No control	Before	Within subjects	Cued recall (words)	Paired associative learning task	10 mins
Jentsch and Wolf (2020) (Germany)	48	23.38 (2.86)	26 F 22 M	20 mins	High	Treadmill	Seated rest watching documentaries	After	Between subjects	Recognition (pictures)	Visuospatial IAPS memory task	24 h
Kao et al. (2018) (US)	36	21.5	18 F 18 M	16 mins	High Moderate	Treadmill	Rest	Before	Within subjects	Free recall (words)	Visual free recall task	Immediate 13 mins
Labban and Etnier (2011) (US)	48	22.02	33 F 15 M	20 mins	Moderate	Cycle ergometer	Rest	Before After	Between subjects	Free recall (story)	Guild Memory Test	35 mins
Labban and Etnier (2018) (US)	15	22.73	10 F 5 M	30 mins	Moderate	Cycle ergometer	Rest	Before After	Within subjects	Free recall and recognition (words)	RAVLT	60 mins
Lind et al., 2019 (Denmark)	81	11.8	33 F 48 M	20 mins	Mod/High Low	Football Football (walking)	Rest	After	RCT	Recognition (pictures)	Visual Memory Task	7 days
Loprinzi et al. (2020a) (US)	23	20.4 (1.3)	65.2% F	15 mins	Moderate	Treadmill	Rest	Before	Within subjects	Free recall (words)	Word list	20 mins
	28	23.1 (5.2)	46.4 % F	0 mins								
	31	21.5 (2.8)	58.1% F	0 mins								
	20	21.5 (10.5)	50% F 50% M	15 mins								
	28	23.1 (50.2)	46.4% F	0 mins								
	73	21 (1.7)	72% F	0 mins								
Loprinzi et al. (2020b) (US)	40	21	47.5% F 52.5% M	15 mins	Moderate	Circuit – body weight (resistance)	Sudoku	Before	Between subjects (2 arm parallel RCT)	Free recall (words, item location)	RAVLT Treasure Hunt Task	Immediate 10 mins
	51	21.7	68.3% F 31.7% M						Between subjects (3 arm parallel RCT)			
Loprinzi et al. (2019b) (US)	80	20.9 (1.2)	61.3% F, 38.7% M	15 mins	Low	Treadmill	Rest withudoku	Before	Between subjects	Free recall (story)	Logical memory task	Immediate 20 mins
	77	21.10 (3.3)	50% F, 50% M	0 mins	High					Free recall (words)	Logical memory task	
	80	21.04 (1.5)	65% F, 35% M	15 mins	High					Free recall (story)	Toronto Word Poll recall	
Loprinzi et al. (2019c) (US)	12 2	21.2	50% F, 50% M	15 mins	Low Moderate	Treadmill	Rest	Before After	Between group	Free recall (story)	Logical Memory Task	45 mins
Ludyga et al. (2018) (Switzerland)	51	21.8 (1.3)	30 F 21 M	15 mins	Moderate	Outdoor running	Reading Task	Before	Crossover, counterbalanced	Free recall (words)	Word list	Immediate 20 mins
McNerney and Radvansky (2015) (US)	13 6	19.22 (1.19)	NK	2 mins	Moderate	Indoor running	Sudoku	Before	Between subjects	Cued recall (words) Recognition (sentence)	Paired Associative learning task	Immediate 1 week
	13 2	19.07 (1.18)						After				
Most et al. (2017) (Australia)	74	19.8	38 F 36 M	5 mins	Moderate	Step ups	Puzzle	Before, After	Between subjects	Cued recall	Paired faces and names	24 h
	80	19.9	40 F 40 M				Finger tapping on lap	After	Between subjects		Paired faces and names	10 mins
	48	19	31 F 17 M				Finger tapping on lap	After	Within subjects		Abstract forms and names	24 mins
	75	21	58 F 17 M				Finger tapping on lap	After	Between subjects		Abstract forms and names	24 mins
Palmer et al. (2019) (Australia)	59	25.75 (8.31)	33 F 28 M	30 mins	Moderate	Stationary cycle	No exercise	Before, After	3 arm RCT	Cued recall (words)	Paired associative learning task	30 mins
	39	22.9 (5.5)	20 F					Before	Between Group			20 mins
Pesce et al., 2009 (Italy)	52	11-12	NK	60 mins	Mod/High	PE class Circuit training	No exercise	Before	Between subjects	Free recall (words)	Visual word list test	2 mins 12 mins
Piepmeier et al. (2020) (US)	29	21.69	29 M 0 F	To exhaustion	Light High	Cycle ergometer	No exercise	Before	Between subjects	Free recall and recognition (words, spatial location)	RAVLT Spatial memory task	Immediate 30 mins 24 h
Pyke et al. (2020) (UK)	19	21.85 (2.43)	11 F 8 M	30 mins	Low, Mod, High	Ergonomic bicycle	Passive rest	After	Within subjects	Recognition (word- image)	Words image pared task	80 mins
	17	19.77 (1.27)	10 F 7 M	30 mins	Moderate		Active rest					90 mins
	23	19.62 (1.51)	20 F 3 M	6 mins	Mod, High		Passive rest					90 mins
Salas et al. (2011) (US)	80	19.35 (2.34)	46 F, 34 M	10 mins	Moderate	Walking	Sitting	Before	Between subjects	Free recall (words)	-	Immediate
Schmidt-Kassow et al. (2014) (Germany)	18	22.8 (2.6)	9 F 9 M	30 mins	Low	Treadmill	Encoding while sitting	During	Within subjects	Cued recall (words)	Auditory paired association task	24 h
	31	21.7 (2.7)	16 F 5 M									
Schramke and Bauer (1997) (US)	96	20 (young) 69 (old)	NK	5-7 mins	Preferred walking pace	Indoor walk	Seated rest	Before	Between subjects	Free, cued recall and recognition (words)	California Verbal Learning Test	20 mins
Siddiqui and Loprinzi (2018) (US)	20	21.1 (1)	8 F 12 M	20 mins	Brisk walking pace (moderate)	Treadmill	Sudoku	Before During	Within subjects	Free recall (words)	Deese-Roediger-McDermott Paradigm	Immediate 25 min
Slutsky-Ganesh et al. (2020) (US)	76	21.6 (3.19)	30.5% M, 69.5% F	20 mins	Moderate	Recumbent bike	No exercise	Before After	Between subject	Free recall (words)	RAVLT	Immediate 24 h
	22	22.76 (3.2)	33.2% M 66.8% F					Before and After				
Sng et al. (2018) (US)	88	23.3 (3.7)	42 F 46 M	15 mins	Preferred walking pace	Treadmill	Relaxation	Before During After	Between subjects	Free, cued recall and recognition (words)	RAVLT	20 mins 24 h
									Prospective memory			
Soga et al. (2017) (Japan)	18	22.3 (2.4)	5 F 13 M	-	Moderate	Cycle Ergometer	Seated rest	During	Within subjects	Recognition (pictures)	Visual picture task	5 mins
Stones and Dawe (1993) (Canada)	20	84.5	17 F 3 M	NK	Low	Stretching	Watch exercise video	Before	Between group	Cued recall (lexical)	Word fluency withemantic/ lexical prompts	Immediate 30 mins
van Dongen et al. (2016) (Netherlands)	72	21.9	48 F 24 M	35 mins	Alternati ve (high low)	Rest	Rest	After	Between group	Cued recall (object location)	Cued recall memory test	48 hours
Wade and Loprinzi (2018) (US)	34	20	17 F 17 M	15 mins	Moderate	Treadmill	Rest	Before	Between subject	Recognition (pictures)	IAPS images task	24 h
Wang et al. (2020) (China)	22	21.6 (3)	0 F 22 M	30 mins	Moderate	Cycle ergometer	Rest	Before After	Within subject	Free recall and recognition (pictures)	Free recall and recognition task	Immediate 1 h 24 h
Weinberg et al. (2014) (US)	46	20	29 F 17 M	-	High	Isometric dynamometer	Rest	After	Within subjects	Recognition (pictures)	IAPS images task	48 h
Winter et al. (2007) (Germany)	27	22.2 (1.7)	27 M 0 F	40 mins (low impact running) 6 mins (sprints at increasing speeds)	High Low	Track	Rest	Before	Within subjects (randomized crossover)	Cued recall (pictures)	Associative vocab learning task	Immediate 1 week 8 months
Yanes and Loprinzi (2018) (US)	40	21	NK	15 mins	Moderate	Treadmill	Sudoku	Before	Between subjects (two armed parallel RCT)	Free recall (paragraph)	Paragraph episodic memory task	20 mins 24 h
Yanes et al. (2019) (US)	24	20.9 (1.8)	12 F 12 M	15 mins	Moderate	Treadmill	Rest	During After	Within subjects (counterbalanced)	Free recall (words)	Word list	-
Zuniga et al. (2019) (US)	30	20.4	20 F 10 M	10 mins	Low	Treadmill	Sitting	Before	Within subjects	Free recall (words) (metacognition)	Word list	5 mins
	57	20	31 F 26 M									

### 3.2 Quantitative analyses

**Figure 4:**
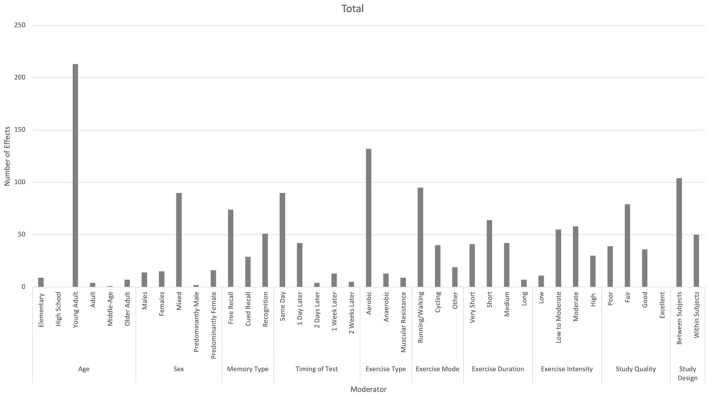
Total number of effects for each moderator.

The total number of effects per primary moderating variable are displayed in Figure 4. Focusing on conditions in which acute bouts of exercise were performed after memory encoding, several moderators influenced long-term episodic memory. Performance differed as a function of participant's age, with young adults showing improved memory performance while the opposite was true for older adults. Our results indicated that older adults' memory performance did not improve before, during, or after acute bouts of exercise.

#### 3.2.1 Exercise before encoding

**Figure 5:**
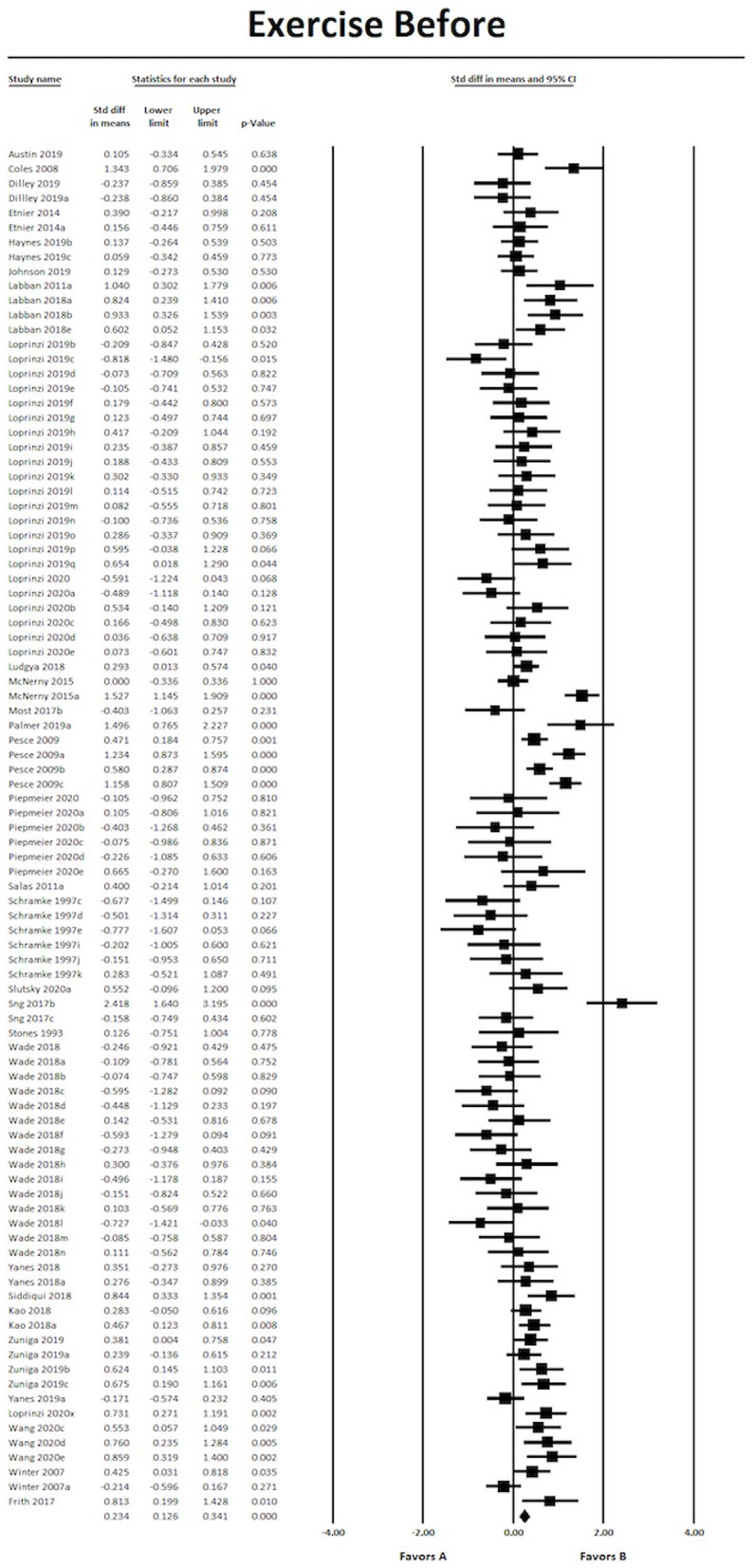
Forest plot indicating effect sizes for exercise before memory encoding.

The overall effect size for exercise before memory encoding compared to control conditions was *d* = 0.23 (95% CI: [0.13, 0.34], *p* < 0.001; Table 2, Figure 5), which constitutes a small effect size. There was evidence of a significant moderating effect for the analysis [Q = 317.17, df (92), *p* < 0.001, I^2^ = 70.81]. For the primary moderator of interest, free-recall (*d* = 0.40, 95% CI: [0.27, 0.53], *p* < 0.001) memory was improved significantly. Cued-recall (*d* = 0.077, 95% CI [−0.267, 0.421]) and recognition memory (*d* = −0.057, 95% CI [−0.207, 0.092]) were not significantly influenced. Analyses of moderators of secondary interest revealed that elementary school children's (*d* = 0.70, 95% CI: [0.37, 1.03], *p* < 0.001) and young adults' (*d* = 0.19, 95% CI: [0.09, 0.30], *p* < 0.001) episodic memory improved, while older adults' (*d* = −0.47, 95% CI: [−0.89, −0.06], *p* = 0.03) memory performance declined significantly. Additionally, significant improvements in episodic memory were detected when mixed-sex samples were measured (*d* = 0.37, 95% CI [0.01, 0.23], *p* < 0.001), memory testing occurred on the same day as encoding (*d* = 0.30, 95% CI [0.17, 0.43], *p* < 0.001), and when exercise parameters included aerobic protocols (*d* = 0.24, 95% CI [0.12, 0.35], *p* < 0.001), cycling modality (*d* = 0.61, 95% CI [0.39, 0.84], *p* < 0.001), medium duration (*d* = 0.64, 95% CI[0.43, 0.85], *p* < 0.001), and moderate intensity (*d* = 0.56, 95% CI [0.38, 0.74], *p* < 0.001). The regression intercept for the Egger's test (intercept 1.90, *p* = 0.002) was statistically significant, indicative of publication bias.

**Table 2:** Moderation results for exercise before memory encoding vs. control.

**Moderator**	**Effect size and precision**		**95% CI**			**Heterogeneity**	
	**Number of ES contributions**	**ES (Cohen's d)**	**Lower CI**	**Upper CI**	***p*** **-value**	**Q-value**	***p*** **-value**
**Age**						20.679	< 0.001
Elementary	6	0.701^***^	0.372	1.030	< 0.001		
Highchool	-	-	-	-	-		
Young adult	80	0.19^***^	0.092	0.295	< 0.001		
Adult	3	1.236	−0.302	2.774	0.115		
Middle-age	-	-	-	-	-		
Older adult	4	−0.474^*^	−0.892	−0.057	0.026		
**Sex**						26.077	< 0.001
Males	11	0.265	−0.014	0.545	0.063		
Females	15	−0.204^*^	−0.379	0.090	0.022		
Mixed	46	0.374^***^	0.005	0.233	< 0.001		
Predominantly male	-	-	-	-	-		
Predominantly female	12	0.178^*^	0.005	0.351	0.044		
**Memory type**						20.564	< 0.001
Free recall	56	0.399^***^	0.267	0.531	< 0.001		
Cued recall	8	0.077	−0.267	0.421	0.660		
Recognition	29	−0.057	−0.207	0.092	0.452		
**Timing of test**						2.969	0.397
Same day	60	0.299^***^	0.171	0.427	< 0.001		
1 day later	19	0.107	−0.134	0.348	0.384		
2 days later	-	-	-	-	-		
1 week later	9	0.101	−0.354	0.556	0.663		
2 weeks later	5	0.116^***^	0.171	0.427	< 0.001		
**Exercise type**						3.479	0.176
Aerobic	82	0.236^***^	0.124	0.349	< 0.001	
Anaerobic	5	0.470	−0.055	0.995	0.079	
Muscular resistance	6	−0.058	−0.400	0.285	0.741	
**Exercise mode**						13.37	0.001
Running/walking	64	0.195^**^	0.066	0.324	0.022		
Cycling	17	0.610^***^	0.385	0.835	< 0.001		
Other	12	0.292	−0.047	0.631	0.091		
**Exercise duration**						22.951	< 0.001
Very short	19	0.200	−0.041	0.440	0.104		
Short	52	0.079	−0.041	0.198	0.198		
Medium	18	0.638^***^	0.430	0.846	< 0.001		
Long	4	0.694^*^	0.056	1.33	0.033		
**Exercise intensity**						24.852	< 0.001
Low	7	0.345^**^	0.138	0.552	0.001		
Low to moderate	36	−0.018	−0.186	0.149	0.832		
Moderate	30	0.563^***^	0.382	0.743	< 0.001		
High	20	0.120	−0.026	0.266	0.106		
**Study quality**						9.212	0.010
Poor	19	0.544^**^	0.309	0.779	< 0.001		
Fair	45	0.129	−0.027	0.285	0.105		
Good	29	0.161^*^	0.016	0.306	0.030		
Excellent							
**Study design**						16.794	< 0.001
Between subjects	66	0.083	−0.055	0.222	0.238		
Within subjects	27	0.506^***^	0.359	0.653	< 0.001		

^*^Indicates statistically significant effect size (*p* < 0.05), ^**^(*p* < 0.01), ^***^(*p* < 0.001).

Reinforcing the findings of the primary search, the secondary search indicated that exercise before encoding led to the largest mnemonic benefits in studies by Etnier et al. (2020), Etnier et al. (2021), McSween et al. (2021), and Schmid et al. (2023).

#### 3.2.2 Exercise after encoding

**Figure 6:**
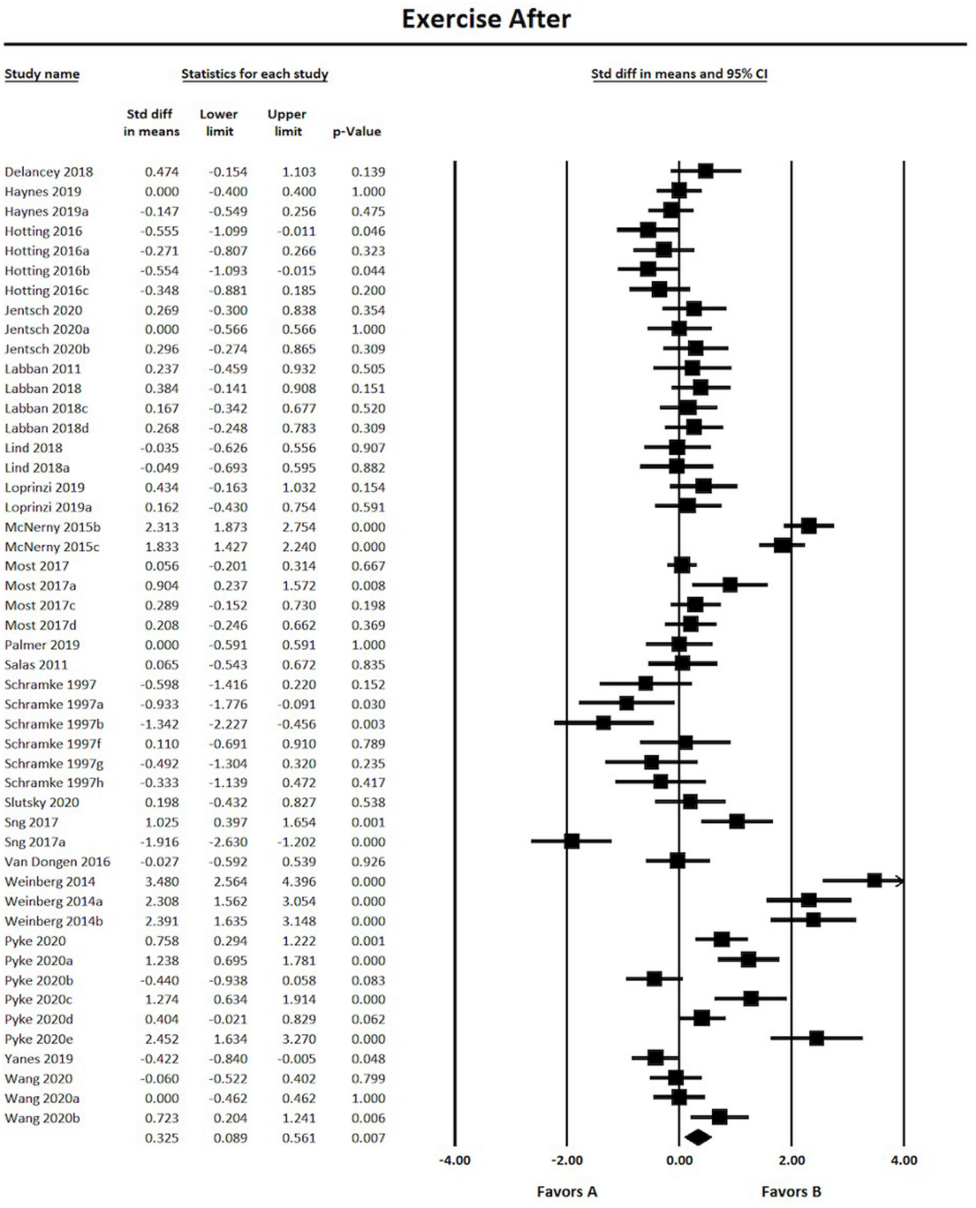
Forest plot indicating effect sizes for exercise after memory encoding.

The overall effect size for exercise bouts performed following memory encoding compared to control conditions was *d* = 0.33 (95% CI [0.89, 0.56], *p* = 0.007; Table 3, Figure 6), which constitutes a small-to-medium effect size. There was evidence of significant moderation effects [Q = 440.37, df (48), *p* < 0.001, I^2^ = 89.10]. For the primary moderator of interest, recognition memory significantly improved when exercise occurred following memory encoding (*d* = 0.623, 95% CI [0.12, 1.13], *p* = 0.015), while free-recall (*d* = 0.194, 95% CI [−0.16, 0.55], *p* = 0.283), and cued-recall (*d* = 0.142, 95% CI [−0.22, 0.51], *p* = 0.448) memory processes were not significantly influenced. Analyses of moderators of secondary interest revealed that young adults' memory function improved (*d* = 0.43, 95% CI [0.18, 0.68], *p* = 0.001) while older adults' episodic memory performance declined (*d* = −0.94, 95% CI [−1.43, −0.45, *p* < 0.001). Other moderators that significantly influenced episodic memory function were detected when samples were predominantly female (*d* = 1.15, 95% CI [0.44, 1.85], *p* = 0.001) and exercise parameters included resistance exercise (*d* = 2.68, 95% CI [1.99, 3.37], *p* < 0.001), long duration exercise (*d* = 2.68, 95% CI [1.99, 3.37], *p* < 0.001), and moderate intensity (*d* = 0.81, 95% CI: [0.43, 1.18], *p* < 0.001). The regression intercept for the Egger's test (intercept 0.71, *p* = 0.67) was not statistically significant, indicating that there was no evidence of publication bias in this set of studies.

**Table 3:** Moderation results for exercise after memory encoding vs. control.

**Moderator**	**Effect size and precision**		**95% CI**			**Heterogeneity**	
	**Number of ES contributions**	**ES (Cohen's d)**	**Lower CI**	**Upper CI**	***p*** **-value**	**Q-value**	***p*** **-value**
**Age**						24.269	< 0.001
Elementary	2	−0.041	−0.477	0.394	0.852		
Highchool	-	-	-	-	-		
Young adult	43	0.427^**^	0.175	0.680	0.001		
Adult	1	0	−0.591	0.591	1		
Middle-age	-	-	-	-	-		
Older adult	3	−0.938^***^	−1.427	−0.449	< 0.001		
**Sex**						5.881	0.118
Males	3	0.207	−0.266	0.681	0.390		
Females	-	-	-	-	-		
Mixed	34	0.259^*^	0.025	0.494	0.030		
Predominately male	-	-	-	-	-		
Predominately female	4	1.146^**^	0.439	1.852	0.001		
**Memory type**						2.534	0.282
Free recall	15	0.194	−0.160	0.549	0.283		
Cued recall	16	0.142	−0.224	0.507	0.448		
Recognition	18	0.623^*^	0.120	1.126	0.015		
**Timing of test**						8.137	0.043
Same day	25	0.136	−0.129	0.401	0.314		
1 day later	16	0.067	−0.166	0.300	0.573		
2 days later	4	2.015^*^	0.442	3.588	0.012		
1 week later	4	1.035	−0.123	2.193	0.080		
2 weeks later	-	-	-	-	-		
**Exercise type**						59.307	< 0.001
Aerobic	41	0.056	−0.113	0.224	0.517	
Anaerobic	5	1.235^**^	0.459	2.011	0.002	
Muscular resistance	3	2.681^***^	1.993	3.369	< 0.001	
**Exercise mode**						7.327	0.026
Running/walking	21	0.058	−0.370	0.486	0.791		
Cycling	21	0.256	−0.003	0.515	0.052		
Other	7	1.312	0.504	2.120	0.001		
**Exercise duration**						54.535	< 0.001
Very short	17	0.439	−0.015	0.894	0.058		
Short	8	−0.041	−0.517	0.435	0.866		
Medium	21	0.058	−0.117	0.233	0.515		
Long	3	2.681^***^	1.993	3.369	< 0.001		
**Exercise intensity**						26.696	< 0.001
Low	2	−0.450^*^	−0.829	−0.071	0.020		
Low to moderate	14	−0.275	−0.620	0.070	0.118		
Moderate	23	0.805^***^	0.431	1.179	< 0.001		
High	10	0.219	−0.114	0.552	0.198		
**Study quality**						27.369	< 0.001
Poor	18	1.061^***^	0.605	1.518	< 0.001		
Fair	24	−0.196	−0.407	0.015	0.068		
Good	7	0.293^**^	0.079	0.506	0.007		
Excellent							
**Study design**						0.100	0.752
Between subjects	33	0.291	−0.059	0.641	0.103		
Within subjects	16	0.363^*^	0.086	0.64	0.010		

^*^Indicates statistically significant effect size (*p* < 0.05), ^**^(*p* < 0.01), ^***^(*p* < 0.001).

Through the secondary search, Loprinzi (2019) found null results, and Zabriskie and Heath (2019) found that exercise during encoding benefitted free recall memory more than exercise before encoding.

#### 3.2.3 Exercise during encoding

**Figure 7:**
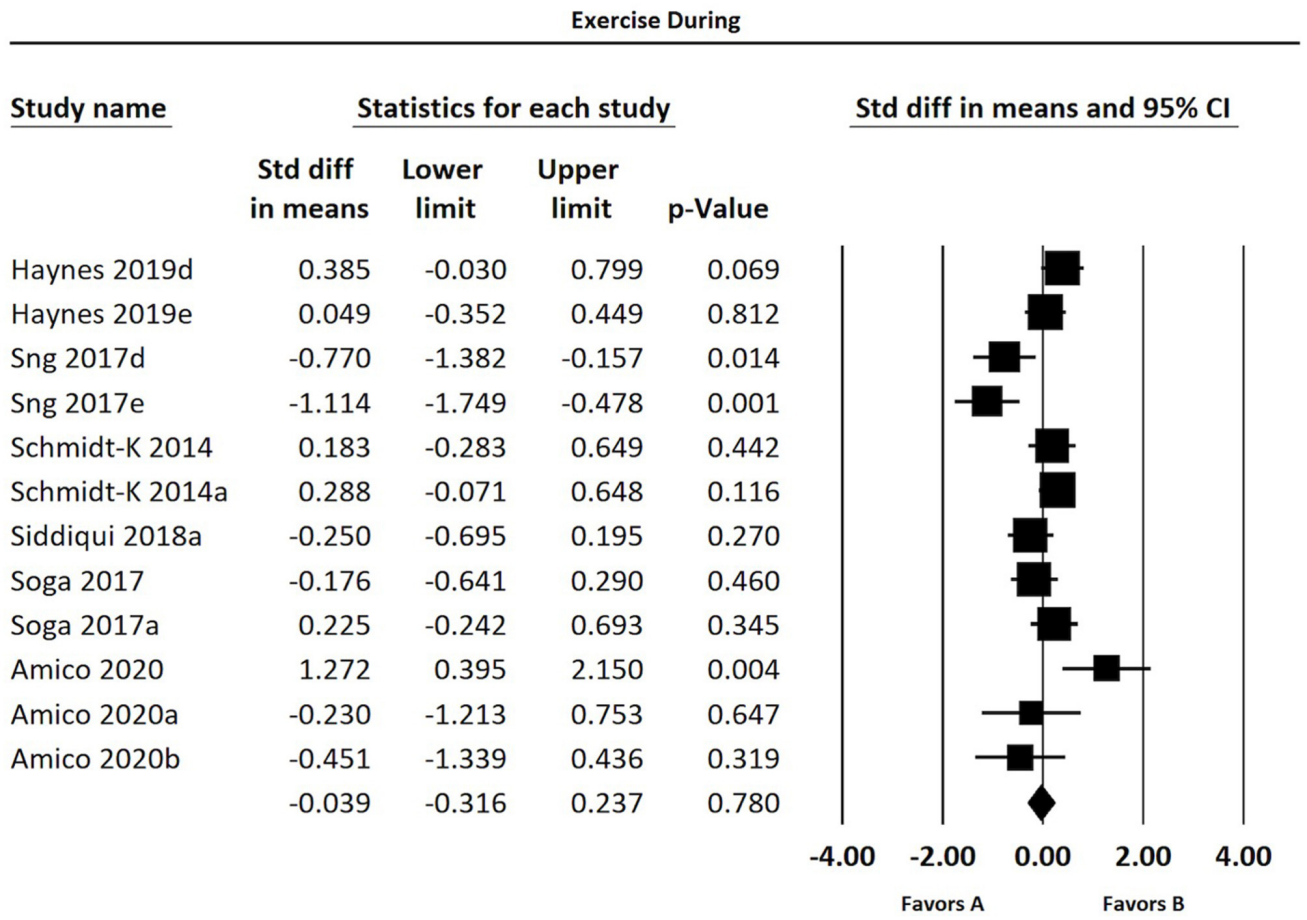
Forest plot indicating effect sizes for exercise during memory encoding.

The overall effect size for exercise performed during memory encoding compared to control conditions was non-significant *d* = −0.04 (95% CI [−0.31, 0.24], *p* = 0.78; Table 4, Figure 7). There was evidence of a significant moderation effect (Q = 36.21, df (11), *p* < 0.001, I^2^ = 69.62). Moderator analyses yielded one significant result. The effect of participants' age on episodic memory was greatest for elementary-aged children (*d* = 1.272, CI [0.395, 2.15], *p* = 0.004). The regression intercept for the Egger's test (intercept −1.62, *p* = 0.40) was not statistically significant, indicating that there was no evidence of publication bias.

**Table 4:** Moderation results for exercise during memory encoding vs. control.

**Moderator**	**Effect size and precision**		**95% CI**			**Heterogeneity**	
	**Number of ES contributions**	**ES (Cohen's d)**	**Lower CI**	**Upper CI**	***p*** **-value**	**Q-value**	***p*** **-value**
**Age**						8.853	0.012
Elementary	1	1.272^**^	0.395	2.15	0.004		
Highchool							
Young adult	10	−0.018	−0.167	0.131	0.453		
Adult							
Middle-age	1	−0.230	−1.213	0.753	0.647		
Older adult							
**Sex**						0.095	0.757
Males	-	-	-	-	-		
Females	-	-	-	-	-		
Mixed	10	−0.057	−0.394	0.28	0.739		
Predominately male	2	0.024	−0.369	0.417	0.904		
**Memory type**						2.207	0.332
Free recall	3	−0.181	−0.814	0.451	0.574		
Cued recall	5	0.232	−0.189	0.653	0.280		
Recognition	4	−0.212	−0.699	0.275	0.394		
**Timing of test**						0.087	0.768
Same day	5	−0.085	−0.451	0.281	0.650		
1 day later	7	−0.224	−0.437	0.439	0.996		
2 days later	-	-	-	-	-		
1 week later	-	-	-	-	-		
2 Weeks later	-	-	-	-	-		
**Exercise type**						0.253	0.615
Aerobic	9	−0.083	−0.365	0.199	0.563	
Anaerobic	3	0.206	−0.885	1.297	0.711	
Muscular resistance						
**Exercise mode**						0.095	0.757
Running/walking	10	−0.057	−0.394	0.28	0.739		
Cycling	2	0.024	−0.369	0.417	0.904		
Other							
**Exercise duration**						1.394	0.498
Very short	5	0.116	−0.376	0.607	0.644		
Short	4	−0.324	−0.970	0.323	0.326		
Medium	3	0.089	−0.235	0.413	0.590		
Long							
**Exercise intensity**						3.572	0.168
Low	2	0.249	−0.035	0.533	0.086		
Low to moderate	5	−0.297	−0.786	0.193	0.234		
Moderate	5	0.116	−0.376	0.607	0.644		
High							
**Study quality**						0.095	0.757
Poor	2	0.024	−0.369	0.417	0.904		
Fair	10	−0.057	−0.279	0.233	0.739		
Good							
Excellent							
**Study design**						1.003	0.317
Between subjects	5	−0.294	−1.075	0.488	0.461		
Within subjects	7	0.115	−0.059	0.290	0.194		

^*^Indicates statistically significant effect size (*p* < 0.05), ^**^(*p* < 0.01), ^***^(*p* < 0.001).

## 4 Discussion

The primary finding of this meta-analytic review is that acute bouts of exercise performed prior to or following encoding have selective effects on episodic memory processes. We assessed 42 primary research studies and our main findings indicated that performance on free-recall tests of memory significantly improved (*d* = 0.399) more than either cued-recall (*d* = 0.077) or recognition (*d* = −0.05) tests of memory when exercise preceded memory encoding. Exercise performed following encoding had a greater impact on recognition memory performance (*d* = 0.623) than on either free- (*d* = 0.194) or cued-recall (*d* = 0.142) performance. A secondary narrative review affirmed the findings of the former meta-analysis. Contemporary psychological theories of memory and recent advances in the study of the neurophysiology of memory systems provide the means to explain why exercise has selective effects on memory processes.

When exercise preceded encoding, there was a significant improvement in memory performance (*d* = 0.29), with the greatest effect exhibited on free-recall processes. Conceptually, free-recall memory is an issue of item remembrance, which can be viewed as the developing strength of a memory trace (Malmberg et al., 2019). Remembering items for later recall is influenced by a wide variety of factors which have been studied extensively. Theories of Levels of Processing (Amico and Schaefer, 2020), strategy utilization (Craik and Lockhart, 1972), mental engagement (Coles and Tomporowski, 2008; Kahneman, 1973; Hockey et al., 1986), and other cognitive constructs have addressed conditions that affect the degree to which individuals allocate attentional resources to environmental events. Physical exercise has also been hypothesized to influence attentional allocation. From the perspective of psychological theory, Audiffren (McMorris et al., 2009) proposed that the energetic property of acute bouts of exercise influences attentional processes and may augment memory storage processes. Energetics theory assumes three interrelated processes: cognition, which reflects an individual's knowledge and skills; affection, which reflects feelings and emotions; and conation, which is the willingness, or motivation, to expend physical and mental effort to perform (Delancey et al., 2019; Haynes and Loprinzi, 2019; Hilgard, 1980; Spirduso et al., 2007). From the neurophysiological perspective, several researchers propose that exercise prior to encoding leads to elevations of brain catecholamines, which presumably increase attention by altering neural signal-to-noise conditions, and by brain-derived neurotrophic factors (BDNF) (Etnier et al., 2014; Hötting et al., 2016; Dilley et al., 2019; Gomez-Pinilla and Hillman, 2013) and growth factors (Loprinzi, 2019). A comprehensive description of brain structures and pathways involved in episodic memory processes and how they may be affected by acute bouts of exercise has been provided by Loprinzi et al. (2017). A meta-analytic review of studies conducted with adults found that moderately intense warm-up exercises had a small positive effect on learning while high-intensity exercise had a substantial negative effect (Johnson et al., 2019; McMorris et al., 2015). The authors of a recent meta-analysis of acute exercise experiments that focused on motor learning drew similar conclusions. Wanner et al. (2020) hypothesize that exercise performed prior to practicing motor tasks influence motor performance by optimizing movement planning, which relies heavily on the allocation of cognitive resources. Psychological theory and neurophysiological research may explain how acute bouts of exercise engage executive processes and control of operations of prefrontal cortical networks that select and weigh the importance of encoded information. Drawing upon the Search of Associative Memory model, exercise prior to encoding appears to alter the sampling and search processes that are involved in free-recall memory. This explanation is in line with those expressed in the contextual-binding model (Yonelinas et al., 2019), which hypothesizes that hippocampal structures are central to item-context associations and activations that influence recollection.

Episodic memory was improved when exercise occurred following encoding (*d* = 0.37). Bouts of exercise enhanced performance on recognition memory tests more than free- or cued-recall tests. Viewed from a psychological perspective, recognition is an issue of item familiarity and involves making a rational response based on the weight of an item and context trace strength via a Bayesian process, which is a reasonable expectation based on past knowledge (Kao et al., 2018; Shiffrin and Steyvers, 1997). Recognition involves discrimination between items that belong to a target population and items that belong to a non-target population (Labban and Etnier, 2011; Green and Swets, 1974). The probability of responding to targets (hits) and responding to non-targets (false alarms) provide the basis for Receiver Operating Curve analyses, which can be applied to the study of recognition memory (Labban and Etnier, 2018; Stanislaw and Todorov, 1999). Studies of attention reveal that the likelihood of hits and false alarms in a given task are affected by a variety of factors (e.g., cost/benefits, ratio of target to non-target items, personal beliefs, and arousal states) (Lind et al., 2019; Warm, 1984). Acute exercise temporarily alters individuals' response bias when performing decision-making tasks (Loprinzi et al., 2021). Exercise-induced shifts in participant's bias would be expected to influence performance on tests of recognition memory. Neurophysiological models of long-term memory propose that item familiarity is altered by emotional arousal and the biological stress response when exercise follows encoding, which is characterized by a cascade of neurohormonal changes (Loprinzi et al., 2019a,b, 2020a,b, 2021; McGaugh, 2000, 2004, 2018). The release of adrenal stress hormones results in widespread changes throughout the peripheral and central nervous system. In particular, the stress hormones epinephrine and cortisol are implicated in memory consolidation. Considerable evidence obtained from animal studies indicates that systemic administration of stimulant drugs, epinephrine, glucose, and cortisol can enhance memory (see reviews by Cahill and McGaugh, 1998; Gold et al., 2001; McGaugh, 2015). Exercise-induced stress responses have also been shown to influence human memory storage (Segal et al., 2012). Much of the consolidation process research has focuses on the bi-directional relation between the amygdala and hippocampus (Loprinzi et al., 2017). The Wanner et al. (2020) review of experiments that focused on motor learning reached similar conclusions. They propose that exercise performed following encoding enhances learning via consolidation and neuroplasticity processes, as opposed to arousal, attention, or energetic processes. Together, psychological theory and neurophysiological research may explain how acute bouts of exercise engage hippocampal processes and the storage of episodic memories. Drawing upon the Retrieving Effectively from Memory model, exercise following encoding appears to impact individual's response bias during recognition memory tests. Drawing on the Contextual-Binding model (Yonelinas et al., 2019), tests of recognition memory involve not only the recognition of an item but also the retrieval of past memory of contexts in which the item was initially acquired. The perirhinal cortex is hypothesized to play the pivotal role for familiarity-based recognition. Brain lesion studies have also provided evidence that the function of the medial temporal lobe is not unitary. Because the medial temporal lobe differentially influences recollection (free-recall) and familiarity (recognition) of items, it provides a dual-process model (Bowles et al., 2010). Our findings are in line with the dual-processing model of the medial temporal lobe as we present a clear distinction between free-recall and recognition memory processes. When exercise follows encoding the consolidation of memory for items may increase the strength of item representation.

Regardless of the temporal relation between exercise and encoding, the strength of the effect of exercise on cued-recall test performance was found to range between very small and small (*d* = 0.077 when exercise preceded encoding; *d* = 0.142 when exercise followed encoding, *d* = 0.232 when exercise occurred during encoding). Cued-recall memory tests are viewed as a hybrid between free-recall and recognition tests (Pesce et al., 2009; Wilson and Criss, 2017; Piepmeier et al., 2020). Free-recall performance depends on self-generated cues and available context cues; recognition performance depends on researcher-generated cues and the participant's available context cues. Cued-recall is similar to free recall in that the individual must generate a target cue that is employed during memory search. Cued-recall is also similar to recognition in that an item cue is provided to the participant. A series of experiments conducted by Wilson and Criss (2017) using cued-recall methods provide data that support predictions from the SAM and REM models. They hypothesize that a cue comes to be associated with a specific target item when a list of cue-target pairs is presented; however, the cue also comes to be associated with other target items on the list. Interference due to mismatches between cues and targets with different trace strengths leads to confusion and results in changes in response bias for memory traces during the search process (Pyke et al., 2020). Prior research using cued-recall methods reveals that test performance is altered via manipulating some list variables, such as word frequency, but not by others, such as context variability (Criss et al., 2011). The findings of the present reviews suggest that acute bouts of exercise performed following encoding has a slightly greater impact on the processes involved in cued-recall test performance than exercise performed prior to encoding. The lack of strong evidence for the relation between exercise and cued-recall memory may be explained via the context-binding model, which hypothesizes that while the pathways that impact recognition and familiarity are distinct, they merge within the medial temporal lobe of the neocortex. The reciprocal relations that exists within neocortical and deeper brain structures result in considerable overlap in the subjective evaluation of memory of past experiences (Eichenbaum et al., 2007).

Since the initial research conducted over a century ago by Yerkes and Dodson (1908), there has been a longstanding interest in the relationship between arousal and memory. The results of the present review suggest that acute bouts of exercise provide an ideal model to advance general theories of memory and learning. Via the systematic manipulation of the temporal relation between exercise and encoding, as well as quantitative and qualitative aspects of exercise, researchers may derive a clearer understanding of brain structures involved in memory as well as psychological and environmental factors that may moderate memory storage and retrieval during recall and recognition tests.

The results of the present review suggest that long-term episodic memory is not influenced by exercise when performed simultaneously with encoding (*d* = −0.04). This finding differs from the conclusion drawn by Loprinzi et al. (2019a), who found that exercise performed under dual-task conditions significantly impaired long-term encoding (*d* = −0.23). The lack of agreement may be due to including the results of several recently published experiments that found that treadmill walking and ergometer cycling (Schramke and Bauer, 1997; Schmidt-Kassow et al., 2014, 2013, 2010) resulted in improvements on tests of long-term memory for words. Alternatively, the lack of clarity may be due to the complexities of dual-task performance and learning. The mental operations that underlie dual-task performance have been studied extensively and several theories have been proposed to explain how individuals optimize decision making when placed in conditions in which they simultaneously perform a motor-movement task (e.g., walking) and a cognitive task (e.g., encoding a word list) (see historical review by Wickens, 2008). Under these conditions, cognitive-motor interference may occur. However, the interference between motoric activities and encoding depends on several factors. A task-classification system developed by Plummer et al. (2013) describes nine dual-task conditions and possible outcomes ranging from no interference, to mutual facilitation, to mutual deterioration, to tradeoffs that differentially impact motor performance and cognitive performance (see reviews by Dietrich and Audiffren, 2011; Tomporowski and Qazi, 2020). Thus, there may be specific dual-task conditions in which the encoding of episodic information into long-term memory may be lessened, enhanced, or have null effects.

A series of experiments conducted recently with children provides insights into dual-task conditions that may facilitate encoding and long-term memory. They may also help explain children's enhanced memory performance found in our meta-analysis. We found that children's memory performance was enhanced when exercise was performed prior to and simultaneously with encoding. These experiments involved teaching children academic material over several training sessions and, as such, were not included in our review. A prototypical experiment conducted by Schmidt et al. (2019) targeted elementary-age children who were instructed to associate foreign animal words with known animal words during four brief teaching sessions that were distributed over 2 weeks. Word pairs were presented to groups of children who repeated the word items while either enacting the movements of the animal, running in place, or sitting at a desk. A cued-recall test administered following the final training session revealed that word recall was greater for children who were physically active during encoding than for children who were inactive. These findings were similar to those obtained in an experiment conducted with pre-school age children (van Dongen et al., 2016; Toumpaniari et al., 2015) and a series of experiments that focused on words acquired during geography, science, and mathematics instruction (Mavilidi et al., 2016, 2017, 2018). In several of these experiments, children's long-term memory gain advantage was maintained for 6 weeks. In a recent meta-analysis affirming these findings, Schmid et al. (2023) report a large, positive relationship between physical activity and episodic free (Hedges g = 0.76) and cued (Hedges g = 0.84) recall memory. Similar to the present review, these results did not translate to recognition memory performance (Hedges g = 0.06).

One explanation advanced for children's memory improvements in these experiments (Paas and Sweller, 2012) was based on Cognitive Load Theory, which makes specific hypotheses concerning the bidirectional relation between working memory and long-term memory stores. The theory posits that the limited operational capacity of working memory is offset by the availability of schemas stored in long-term memory. Schemas, which consist of multiple items, reduce the computational limitations of working memory. Paas and Sweller (2012) and Paas and Ayres (2014) draw on an evolutionary theory of human cognitive architecture (Geary, 2006), and suggest that schemas controlling motor movements take precedence over schemas that organize and refine the acquisition of semantic information. However, motor schemas can be used to leverage the encoding of secondary, academic material.

An alternative explanation for these findings (Mavilidi et al., 2021) is grounded in ecological theories of embodiment, which propose that learning emerges from a dynamical interaction among an individual's body movements, the sensory experiences obtained from the movements, and the context of those movements (Lindgren and Johnson-Glenberg, 2013; Wade and Whiting, 2011; Newell, 1986). Memory of movements accumulates in real time via sensory and motor experiences obtained during physical actions that include gestures, walking, and play (Gallagher and Lindgren, 2015). Movements that occur during enactment are hypothesized to engage not only the motor system but also to facilitate the construction of mental representations that enhance memory recall (Cappuccio, 2019).

Several researchers have suggested that acute bouts of exercise may be particularly useful for enhancing cognition in developing children (Tomporowski et al., 2008; Howie and Pate, 2012; Donnelly et al., 2016). This conclusion should be taken with caution as our analyses were based on the results of a limited sample of experiments. If substantiated, the finding would be of importance to educators who could implicate exercise interventions in school setting to improve children's cognitive function.

### 4.1 Analyses of moderators of secondary interest

The analyses revealed commonalities and differences with prior systematic reviews. Focusing on conditions in which exercise was performed prior to encoding, participants' age was found to influence long-term memory. Young adults had significantly better memory performance compared to older adults' performance. These results are in line with those of Roig et al. (2013) and Loprinzi et al. (2019a). The delay between encoding and subsequent tests of memory also influenced the strength of the relation. Performance on memory tests completed during the same day and within 24 h later of encoding was significantly better when compared to longer delays, with the exception of one experiment that evidenced enhanced memory 2 weeks after encoding. Exercise intensity influenced memory performance at low and moderate levels, but not high intensity levels (Roig et al., 2013). These findings differ from those of Loprinzi et al. (2019a), who found high intensity exercise moderated long-term memory. Exercise mode also influenced memory performance, with aerobic exercise producing greater effects than anaerobic and resistance exercise. Exercise duration also impacted the relation, with medium to long durations producing greater effects than shorter durations. Cycling and running/walking impacted memory more than other activities (Loprinzi et al., 2019a).

Meta-analytic evidence supports the notion that chronic exercise interventions improve long-term memory (Northey et al., 2018) and executive function (Chen et al., 2020) among older adults. Additional evidence is needed to definitively clarify the relationship between acute exercise and long-term memory among older adults in clinical and applied settings. Children did not benefit from exercise under these encoding conditions. The delay between encoding and memory tests influenced the relation, with larger effects obtained when measured within 48 h and 1 week following encoding than following shorter delays. It may be that the underlying brain structures involved in consolidation processes may lead to more durable episodic memory storage than the activation of prefrontal neural circuits produced by exercise performed prior to encoding. Exercise intensity performed at low- and moderate-levels resulted in larger positive effects than high intensity exercise, and exercise durations that were relatively long produced greater effects than shorter periods. Exercise type also moderated the relation, with anaerobic and resistance training producing greater effects than aerobic exercise. These results differ from those obtained under conditions in which exercise was performed prior to encoding. While these findings may reflect the limited number of effects available for analyses, they highlight an important variable in need of further examination. The role of exercise mode also differed for this analysis; cycling and other modes were superior to running/walking. The evidence for the moderating effects of specific variables on episodic memory obtained in this and prior reviews (Roig et al., 2013; Loprinzi et al., 2019a) provide numerous avenues for future research that explores the relation between exercise and cognition. The characteristics of the exercise modality impacted the relationship between exercise and long-term memory, regardless of the temporal relation. Given the range of exercise characteristics that were analyzed, the possibility of variability was highly likely, potentially impacting the interpretation of the results. The most common characteristics of exercise modalities in the present meta-analysis consisted of short, low to moderate intensity aerobic exercise protocols that were short in duration. The variation in exercise modalities often has been implicated as a potential mediator between the exercise-cognition relationship (Tomporowski and Pesce, 2019). Though the heterogeneity among exercise protocols may introduce some variability that makes interpretation of results difficult, it is evident that most randomized controlled trials utilize similar exercise protocols (Figure 4).

### 4.2 Limitations and future directions

The primary finding of this review is that the strength of the exercise-memory relation depends on the type of memory process measured. It is acknowledged, however, that the results obtained were restricted to episodic long-term memory, which is only one of several types of memory processing. Additional research designed to understand how the interactions among multiple types of long-term memory processes (e.g., semantic, spatial, perceptual, and procedural) is needed. There are research areas that necessitate additional scrutiny. For example, the analysis of experiments in which participants exercised simultaneously while encoding yielded null effects. Yet, experiments conducted with children that involved multiple training sessions consistently reported memory gains for those who were physically active while encoding academic material. The duration of each lesson and the length of the instructional interventions were brief and it is unlikely that the physical activity induced the changes in brain structure and function found with chronic exercise interventions (McMorris, 2016b; Voss et al., 2011). In the main, researchers have made distinctions between acute, single-bout experiments and chronic exercise interventions lasting months. Chronic exercise experiments typically use pre- and post-treatment performance to assess the effects of the intervention. Relatively little is known about changes in the strength of memory traces that may occur over multiple training sessions. Few experiments that manipulate exercise provide information concerning the effects of repetition and practice on participant's long-term memory over repeated training sessions.

The selection of a single theory of long-term episodic memory (i.e., Atkinson and Shiffrin, 1968, 1971) to serve as a framework for the present review is a limitation. Episodic memory is a complex phenomenon that has attracted the attention of researchers for over 150 years. Numerous psychological theories have been proposed to explain the processes that underlie memory. Considerable theory-based research has been performed to address the variables that influence free-recall, cued-recall, and recognition memory (Piepmeier et al., 2020; Criss and Howard, 2015). The intent of the present review was not to test specific theories or models but, rather, to determine whether the effects of acute bouts of exercise have global or selective effects on episodic memory. The results of our review may stimulate additional research that derive hypotheses from psychological theories as well as from neurophysiological models proposed by Yonelinas et al. (2019) to explain how the context in which items are encoded influenced item storage, recall, and recognition.

Additional well-designed exercise experiments that explore conditions that promote memory encoding and retention are warranted. The assessment of the quality of the experiments selected for this review revealed that the majority were rated as poor or fair. The quality of such research can be bolstered by utilizing reporting guidelines as a method of best practice and by conducting experiments in which hypotheses are grounded in established psychological theory and confirmed by convergent evidence obtained from studies that explore the neurobiology of memory.

Our analyses for the effects of exercise prior to encoding revealed a significant regression intercept for the Egger's test, suggesting evidence of publication bias as a limitation. Effects of exercise after and during encoding did not reveal a significant regression intercept for the Egger's test. Future well-conducted experiments with limited publication bias are warranted to strengthen the current exercise-cognition literature.

Advances in exercise research that focus on moderators that influence the relation between exercise and long-term declarative memory will benefit academics who seek to explain the phenomenon as well as practitioners who plan interventions designed to influence cognitive functions. The results of our review are germane for translational research and application. Physical activity and exercise breaks during children's academic routines are often recommended as interventions that favorably impact class performance and learning (Tomporowski et al., 2015). The results of experiments that examine the effects of exercise on students' academic performance, however, are quite variable (see reviews by Donnelly et al., 2016; Daly-Smith et al., 2018; Singh et al., 2019). The discrepancies that exist across experiments that focus on academic progress may be explained, at least partially, by the temporal relation between physical activity and academic classwork and by the methods that are used to assess learning.

## 5 Conclusions

The present review and systematic meta-analysis focused on mental processes that underlie the encoding and retrieval of information from long-term episodic declarative memory as assessed by tests of free-recall, cued-recall, and recognition. Based on a contemporary theory of long-term memory, we provide evidence that bouts of exercise differentially influenced free-recall and recognition memory processes. Exercise performed prior to encoding influence memory search processes that are employed in free-recall memory tests but not cue-recall or recognition memory. Exercise may alter free-recall processes via mechanisms that impact working memory and attentional allocation policy. Exercise performed following encoding influence memory search processes that are employed during recognition memory and to a lesser degree cued-recall, but not free-recall. Exercise may alter the consolidation of episodic memory. These results provide a theory-based approach to predicting the effectiveness of exercise interventions designed to enhance memory storage and retrieval. Left to be disentangled are the research outcomes obtained from studies of episodic memory that examine the effects of exercise performed simultaneously with encoding. We obtained tantalizing evidence that young children may be impacted via properly designed dual-task academic interventions. Additional dual-task research may lead to the development of exercise interventions designed for children in academic settings as well as older adults who evidence degraded memory function.

## Data availability statement

The raw data supporting the conclusions of this article will be made available by the authors, without undue reservation.

## Author contributions

AQ: Writing – original draft, Writing – review & editing. DS: Writing – review & editing. NG: Writing – original draft, Writing – review & editing. KL: Writing – original draft, Writing – review & editing. AD-S: Writing – original draft, Writing – review & editing. PT: Writing – original draft, Writing – review & editing.
