# Supplementary figures and images for: The effects of acute exercise on long-term episodic memory: a systematic review and meta-analysis
Source: Front Cognit. 2024 Apr 4;3:1367569. doi: 10.3389/fcogn.2024.1367569 (PMC13281107; doi:10.3389/fcogn.2024.1367569)

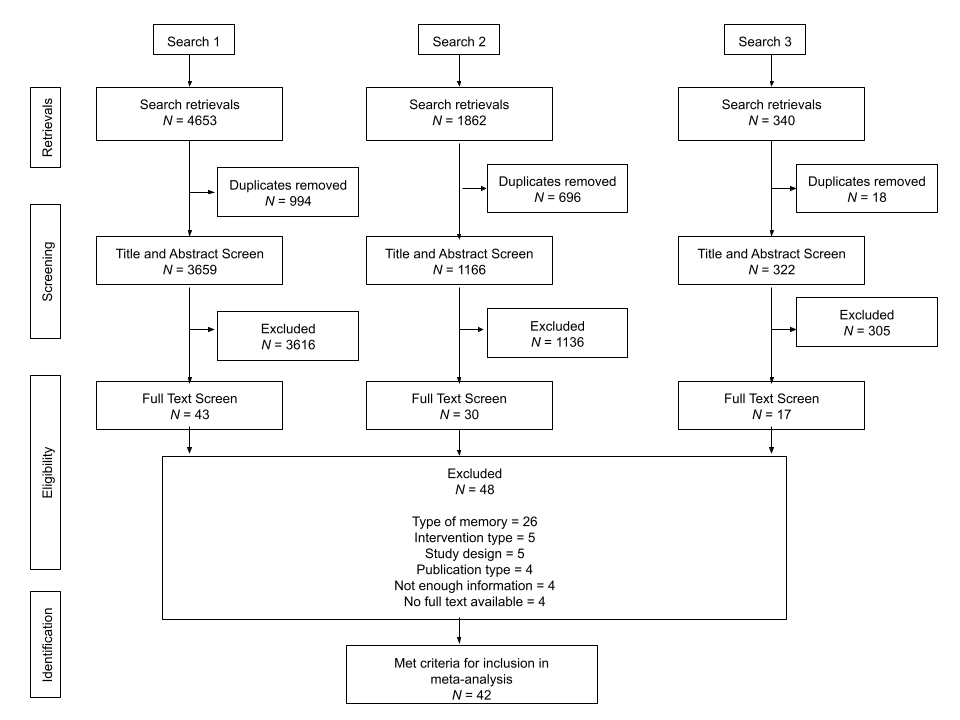


**Figure S1**. Detailed flow diagram illustrating retrieval and inclusion in meta-analysis

Supplement: Supplementary file 1 [file Data_Sheet_1.docx]
